# Advancing Therapeutic Strategies for Nonsense‐Related Diseases: From Small Molecules to Nucleic Acid‐Based Innovations

**DOI:** 10.1002/iub.70027

**Published:** 2025-05-27

**Authors:** Davide Ricci, Ilenia Cruciata, Ignazio Fiduccia, Emanuele Vitale, Federica Corrao, Alessio Branchini, Pietro Salvatore Carollo, Ivana Pibiri, Laura Lentini

**Affiliations:** ^1^ Department of Biological, Chemical and Pharmaceutical Sciences and Technologies University of Palermo Palermo Italy; ^2^ Department of Life Sciences and Biotechnology and LTTA Centre University of Ferrara Ferrara Italy

**Keywords:** gene editing, genetic rare diseases, mRNA‐based therapy, nonsense mutations, nonsense‐mediated decay, premature termination codon, translational readthrough

## Abstract

Nonsense mutations in gene coding regions introduce an in‐frame premature termination codon (PTC) in the mRNA transcript, resulting in the early termination of translation and the production of a truncated, nonfunctional protein. The absence of protein expression and the consequent loss of essential cellular functions are responsible for the severe phenotypes in the so‐called genetic nonsense‐related diseases (NRDs), such as cystic fibrosis, hemophilia, Duchenne muscular dystrophy, Fabry disease, Choroideremia, Usher syndrome, Shwachman–Diamond syndrome, and even certain types of cancer. Nonsense mutations pose a significant challenge in the treatment of NRDs, as a specific approach directly addressing this genetic defect is currently unavailable. Developing new therapeutic strategies for nonsense suppression is a crucial goal of precision medicine. This review describes some of the most promising therapeutic approaches and emerging strategies for treating NRDs. It considered both the use of small molecules to interfere with molecular mechanisms related to nonsense mutations, such as translational readthrough‐inducing drugs (TRIDs) or inhibitors of the nonsense‐mediated decay (NMD) pathway, and also innovative approaches involving nucleic acids, such as gene editing, anticodon engineered‐tRNA (ACE‐tRNA), or mRNA‐based therapy. Future research should focus on refining these approaches and exploring integrated and personalized treatments to enhance therapeutic outcomes and ensure continuous improvement in the quality of care.

**Abbreviations**
NMD: nonsense‐mediated decay
NRDs: nonsense‐related diseases
NTC: natural termination codon
PTC: premature termination codon
TR: translational readthrough
TRIDs: translational readthrough‐inducing drugs

## 1 Introduction

Nonsense mutations, also known as stop mutations, are alterations in the DNA sequence that affect gene coding regions, causing the introduction of an in‐frame premature termination codon (PTC) in the mRNA sequence. This occurs when a sense codon is converted into a nonsense codon. A PTC in the mRNA transcript results in the premature termination of protein synthesis, as the ribosomal translation process stops earlier than usual, producing a shortened, nonfunctional polypeptide. Usually, an open reading frame (ORF) is delimited between an AUG codon as the initiation signal of the translation process and one of the three stop codons, that is, UGA, UAA, and UAG (*opal*, *ochre*, and *amber*, respectively), which represent the natural termination codons (NTCs). During the normal mRNA translation, the entry of the NTC in the A‐site of the ribosome recruits translation termination factors, such as eRF1 (eukaryotic Release Factor 1), allowing the detachment of the ribosomal machinery and the newly synthesized protein.

Single‐nucleotide substitutions, frameshifts, and splicing mutations in the DNA coding region that alter the nucleotide sequence within the ORF can lead to the formation of a PTC, resulting in the early termination of translation and the production of a truncated and nonfunctional protein.

Truncated proteins generated from nonsense mutations are potentially hazardous to the cell, and two intracellular quality control mechanisms prevent their accumulation. At the mRNA level, PTC‐bearing mRNAs are selectively degraded by the nonsense‐mediated decay (NMD) machinery [1]. At the post‐translational level, aberrant proteins are selectively recognized and processed by compartment‐specific quality control pathways. For instance, proteins within the secretory pathway are monitored by the endoplasmic reticulum quality control system, which targets misfolded species for proteasome‐mediated degradation [2].

The absence of protein expression and the resulting loss of function due to a nonsense mutation generate severe phenotypes in the so‐called nonsense‐related diseases (NRDs).

Indeed, nonsense mutations represent about 10% of all the genetic mutations associated with human‐inherited diseases [3], and approximately 5%–70% of individual cases of most genetic disorders are linked to nonsense mutations [4]. NRDs are related not only to prevalent diseases such as cancer but also to rare and ultra‐rare diseases such as cystic fibrosis (CF), hemophilia, Duchenne muscular dystrophy, Fabry disease, choroideremia, Usher syndrome, Shwachman‐Diamond syndrome, and epidermolysis bullosa.

There is no specific therapeutic approach that directly targets NRDs. Developing new strategies to address these conditions is a critical goal in precision medicine. Such approaches could enable treatment not only for prevalent diseases—often the primary focus of therapeutic research—but also for rare and ultra‐rare conditions by developing technologies that target common underlying genetic defects.

In recent years, research in this field has garnered considerable attention, and numerous therapeutic approaches are being developed to treat nonsense mutations. These approaches ultimately aim to restore protein expression and cellular function.

This review presents some of the most promising existing therapeutic approaches and emerging strategies for treating NRDs. It reports state‐of‐the‐art findings in this field and considers completed and ongoing clinical trials with novel treatments.

## 2 Therapeutic Approaches Against Nonsense: Where Are We Now?

### 2.1 Translational Readthrough‐Inducing Drugs (TRIDs)

Translational readthrough (TR) involves the post‐transcriptional suppression of a stop codon due to the mispairing of it with a near‐cognate tRNA rather than the eukaryotic release factor 1 (eRF1) [5]. TR physiologically occurs at a basal level with NTCs at a rate ranging between 0.001% and 0.1%, which can reach 0.1%–1% with a PTC restoring, in the last case, the production of a full‐length protein [3, 6]. TR can also be pharmacologically induced at a higher rate through the use of a particular class of drugs known as TRIDs, which open new horizons in the treatment of nonsense mutations.

**FIGURE 1:**
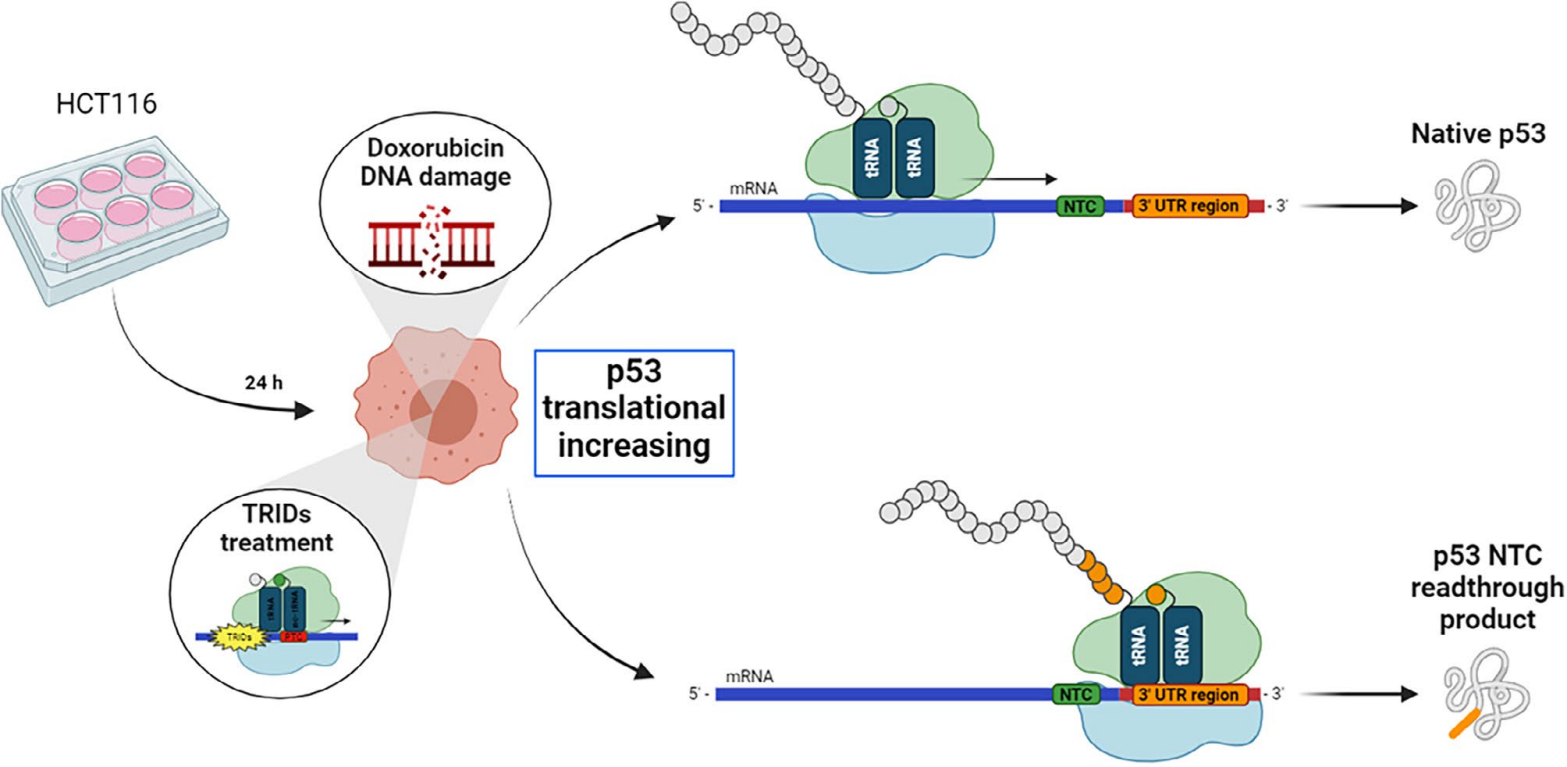
TRID molecules can induce translational readthrough of natural mRNA termination codons (NTCs), including those at the p53 NTC. This miscoding error could alter protein expression, nuclear localization, and/or DNA‐protein interactions [10].

Aminoglycoside antibiotics have been the first molecules primarily studied as T‐inducing compounds on nonsense mutations since Howard and colleagues succeeded in the suppression of nonsense mutations in the *CFTR* gene by using geneticin (G418), thus producing full‐length functional proteins in a CF model system [7]. Furthermore, Floquet and colleagues demonstrated that treating primary breast carcinoma cells HDQ‐P1 (harboring the p. R213X mutation) with the aminoglycoside G418 rescued both p53 transcripts and protein levels, compared to the untreated condition [8]. Later, by treating HDQ‐P1 cells with G418 and the MDM2 inhibitor nutlin 3a, Zhang et al. demonstrated an enhancement of p53 at both the transcript and protein levels, which correlated with significantly increased mRNA levels of a set of p53 target genes [9]. In addition, we showed that NV TRID molecules do not promote readthrough of the NTCs (Figure 1) [10].

The TR effect of aminoglycosides is due to their ability to bind the eukaryotic ribosomal decoding center, specifically interacting with the 18S rRNA. This induces conformational changes that reduce the fidelity of codon recognition, resulting in the insertion of a near‐cognate amino acid at the PTC site and allowing translation to continue, thereby producing a full‐length protein.

However, this mechanism can also affect NTCs, highlighting a lack of selectivity for PTCs and potentially leading to harmful off‐target effects [11].

Moreover, the clinical use of aminoglycosides at the concentrations and durations required for therapeutic benefit is limited by significant adverse effects, most notably hearing loss and nephrotoxicity, which are unrelated to their TR‐inducing activity [12].

Recently, Bidou and colleagues identified NB124 (also known as ELX‐02), a new‐generation aminoglycoside that exhibits high TR activity in the p. R213X mutation [13]. Specifically, treatment of HDQ‐P1 cells with NB124 stabilized p53 mRNA and increased the synthesis of full‐length p53 protein. The rescued p53 protein also increased the mRNA levels of its target genes, BAX and p21. Moreover, the increased synthesis of full‐length p53 protein triggered apoptosis in the H1299 cell line, a lung carcinoma cell line stably expressing a p53 construct harboring the p. R213X mutation [13]. In this context, the synthetic aminoglycoside NB124 has been designed to enhance readthrough and reduce unwanted side effects. It lacks characteristic aminoglycoside toxicity in chronic treatment. It underwent Phase I clinical trials, including single and multiple‐ascending doses, in healthy volunteers, resulting in a well‐tolerated treatment with no adverse effects [14, 15]. In 2023, Eloxx Pharmaceutical announced the completion of a Phase II clinical trial for treating patients with CF and Alport syndrome using ELX‐02, reporting significant improvements in patient conditions following treatment in both cases [Eloxx Pharmaceuticals Reports]. In particular, data evaluation for CF patients reveals an enhancement in lung function, specifically in the percent predicted forced expiratory volume (ppFEV1) (NCT04135495). In Alport Syndrome patients, kidney biopsies from all three patients treated with ELX‐02 showed improvements in podocyte foot process effacement, a key indicator of kidney health, leading to the start of Phase III clinical trials (NCT05448755). Additionally, ELX‐02 has recently been tested for the treatment of Coagulation Factor V deficiency caused by nonsense mutations. The treatment was demonstrated to restore protein expression in a dose‐dependent manner in both in vitro and ex vivo reporter systems [16].

Aminoglycosides and their derivatives are not the only molecules that fit in the TRIDs family. In this regard, a luciferase‐based high‐throughput screening (HTS) of suitable candidates (> 800,000 small molecules) led to the identification of a non‐aminoglycoside compound with readthrough‐inducing activity, called PTC124, also known as Ataluren, developed by PTC Therapeutics [17, 18, 19]. PTC124 is a diaryl‐1,2,4‐oxadiazole with no structural resemblance to aminoglycosides, and it has been proven to be less toxic than them [20]. Due to its features, it has been regarded as a promising compound for treating genetic disorders caused by nonsense mutations, particularly those presenting the *opal* (UGA) mutation. Ataluren underwent various clinical trials for CF; however, it failed to achieve the predefined endpoints in Phase III, despite potential bias due to reproducibility issues in determining potential differences across multiple sites and a high number of false positives [21, 22]. In contrast, Duchenne muscular dystrophy (DMD) clinical trials yielded positive results, leading to its approval as a drug. It was the only drug, commercially known as Translarna, conditionally approved by the European Union for nonsense‐derived DMD treatment. The use has been guaranteed until January 2025, when the Committee for Medicinal Products for Human Use (CHMP) concluded that the benefit–risk balance of Translarna was negative. Consequently, the marketing authorization for Translarna was not renewed in the European Union. This decision is under discussion (Meeting highlights from the Committee for Medicinal Products for Human Use (CHMP), 22–25 January 2024 [23]; 14–17 October 2024 [24]) (Table 1).

**TABLE 1:** Comprehensive analyses of target, mechanism of action, toxicity, and clinical trials for each readthrough agent analyzed.

Molecule	Target	Mechanism of action	Toxicity	Clinical trial
G418_[33]_	Ribosome A site	Proofreading activity inhibition	Nephrotoxicity Ototoxicity	NCT00458341 NCT00004452
PTC124_[48]_	Ribosome	Mispairing tRNA	Low toxicity	NCT02107859 NCT00264888
ELX‐02_[37]_	Ribosome A site	Proofreading activity inhibition	Low toxicity	NCT04537793 NCT04126473 NCT04135495 NCT05448755
NV848, NV914, NV930_[13]_	Ribosome	Mispairing tRNA	Low toxicity	N/A
2,6‐diaminopurine_[35]_	Ribosome	Mispairing tRNA	Low toxicity	N/A
SRI‐41315_[60]_	eRF1	Enhance eRF1 degradation	Low toxicity	N/A
5‐Fluoruridine_[53]_	mRNA	Nucleoside analogs insertion	Cytotoxic effect	N/A
RTC13/RTC14_[44]_	Ribosome	Mispairing tRNA/NMD inhibition	Low toxicity	N/A
Clitocine_[24]_	mRNA	Mispairing tRNA	Moderate toxicity	N/A

Recently, PTC124 has been administered to an infant with D‐bifunctional protein (DBP) deficiency, a life‐threatening autosomal recessive peroxisomal enzyme disorder characterized by hypotonia, seizures, craniofacial abnormalities, psychomotor delay, deafness, and blindness in the neonatal period, with death typically occurring by the age of two. After a two‐year treatment period, the patient showed improved swallowing and progressive motor and speech development [25].

PTC414, an analogue of PTC124, was evaluated for its ability to target nonsense mutations and restore Rab Escort Protein 1 (REP1) function in both primary human fibroblasts and a zebrafish model of choroideremia, demonstrating greater effectiveness and improved pharmacokinetic characteristics when compared to PTC124. However, it is worth noting that in the zebrafish model, PTC414 showed some nephrotoxicity, which was not observed with PTC124. Moreover, compared to the zebrafish model, *CHM* ^*Y42X/y*^ fibroblasts did not show a significant increase in REP1 rescue, although there was an increase in prenylation activity [26]. Furthermore, Sarkar and colleagues demonstrated that PTC124 treatment of CHM K258X fibroblasts did not rescue protein expression [27].

New 1,2,4‐oxadiazoles have been designed, sharing the oxadiazole core with Ataluren. These novel small molecules, called NV848, NV914, and NV930, demonstrated high readthrough activity by producing a full‐length, functional CFTR protein either from mutant *CFTR* cDNAs with UGA stop mutations as well as in a cell model of CF [28, 29], without impacting NTCs [10]. The dose–response analyses revealed that all three molecules had more significant activity than Ataluren [29]. Moreover, an acute toxicity study in a murine model suggested that the three molecules exhibited good tolerability and a low health risk, classifying NV848 and NV930 as category 4 and NV914 as category 5 under the Globally Harmonized System (GHS) of Classification and Labeling of Chemicals [30]. NV848, in particular, was tested for its readthrough activity and efficacy in vivo, demonstrating the ability to rescue CFTR protein expression after chronic treatment in a mouse model carrying the G542X nonsense mutation in the *CFTR* gene [31]. The same molecule also demonstrated its ability to restore SBDS protein expression in both lymphoblastoid cell lines and periodontal ligament stem cells from Shwachman–Diamond syndrome patients carrying the K62X nonsense mutation in the *SBDS* gene [32].

By HTS, other NV compounds, NV2445, NV2899, NV2907, NV2909, and NV2913 have been developed. They exhibit low cytotoxicity and promote the expression of CFTR protein in transfected HeLa cells with the plasmid for the UGA stop mutation [33].

Other novel non‐aminoglycoside compounds have been brought to attention by a protein transcription/translation (PTT)‐ELISA assay, screening nearly 34,000 compounds. These new therapeutic targets for readthrough compounds, designated RTC13 and RTC14, have been tested in DMD mice (mdx) via intramuscular injection. Only RTC13 was shown to partly restore dystrophin protein in various muscle types and improve muscle function. At the same time, RTC14 was unable to promote efficient readthrough activity compared to RTC13 and PTC124 [34].

Amlexanox is another compound worthy of note for its ability to promote PTC readthrough, an anti‐allergic and anti‐inflammatory drug that has been on the market for more than 30 years [35, 36]. Scientists have studied its ability to inhibit NMD and promote the readthrough of PTCs. Still, they have stated that Amlexanox is not particularly effective in restoring a full‐length protein, even when combined with other molecules [37].

2,6‐Diaminopurine (DAP), a purine derivative, is another compound known to induce TR. It has been found to enhance the readthrough of the PTC UGA in the *TP53* gene within cancer cells, inhibiting the activity of the FtsJ RNA 2′‐O‐Methyltransferase 1 (FTSJ1). Tests have shown that DAP exhibits very low toxicity and high efficacy [38]. DAP has been successfully tested on mice, organoids derived from murine or patient cells, and in cells from CF patients, resulting in the ability to restore the expression and functionality of the nonsense‐mutated *CFTR* gene, which presents UGA as a PTC. In addition, DAP resulted in very stable plasma levels and was well distributed throughout the body. Thanks to its pharmacokinetic properties, it was also administered to mice through breastfeeding [39].

In general, interest in TRIDs is higher than ever, as evidenced by the growing number of published compounds that demonstrate readthrough activity in vitro and are now advancing into preclinical development, such as SRI‐41315, 5‐fluorouridine, and Clitocine [40, 41, 42].

**FIGURE 2:**
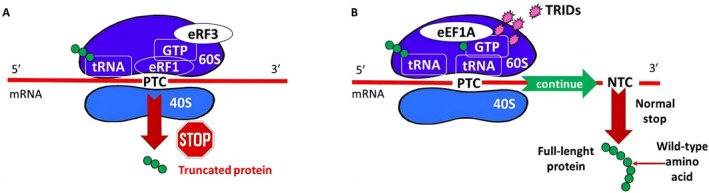
(A) Premature termination translation of a PTC‐bearing mRNA, synthesizing a truncated protein. (B) Translational readthrough of a PTC induced by TRIDs [43].

These studies demonstrate that researchers are actively exploring the study of TRIDs to enhance the efficacy of these molecules, both alone and in co‐administration, thereby moving toward a new frontier in personalized medicine (Figure 2).

### 2.2 Nonsense‐Mediated mRNA Decay (NMD) Inhibition: A Target for Translational Readthrough Amplification

Nonsense‐mediated mRNA decay (NMD) is a crucial quality control mechanism that ensures the integrity of nascent transcripts by detecting and degrading erroneous mRNAs. NMD activity significantly influences the mRNA pool available for TR, thus affecting the efficacy of its pharmacological induction by TRIDs [44]. NMD primarily targets PTCs‐containing mRNAs, which may arise due to nonsense mutations or splicing aberrations, such as intron retention or exon skipping. This mechanism regulates approximately 10% of the transcriptome [45]. NMD is activated in the nucleus and cytoplasm when a PTC is detected. Typically, a PTC is located upstream of the last exon and at least 50 nucleotides from the penultimate exon‐exon junction. A PTC prevents ribosomal progression, leading to the exon junction complex (EJC) retention, which marks the transcript for degradation [46, 47].

The “50‐55 nt rule” helps predict NMD activation in about 50% of known cancer‐related gene mutations, with accuracy increasing to 75% when additional non‐canonical rules are considered. The efficacy of NMD activation decreases under three conditions: (1) when the PTC is within the first 150 nucleotides of the 5′‐end, (2) when the PTC is located in a long exon (≥ 400 nucleotides) far from an exon‐exon junction, and (3) when the transcript has a short half‐life due to competing degradation pathways [45]. Although the exact mechanism of NMD activation remains unclear, a widely accepted model suggests that the ribosome stalling at a PTC disrupts the interaction between eukaryotic release factor 3 (eRF3) and poly(A)‐binding protein (PABP). Normally, eRF3 interacts with PABP at the NTC, ensuring efficient termination. However, this interaction is impaired at a PTC, decreasing translational termination efficiency and triggering NMD. This explains why TR levels are naturally higher in nonsense‐mutated genes (> 1%) compared to non‐mutated ones (< 0.1%) [44, 48]. The NMD pathway relies on seven core factors: UPF1, UPF2, UPF3, SMG1, SMG5, SMG6, and SMG7. UPF3 binds the EJC in the nucleus during splicing, and upon cytoplasmic export, UPF2 associates with UPF3 to form a heterodimer. During ribosome stalling, eRF1 and eRF3 recruit UPF1, which interacts with SMG1, forming the SURF complex (eRF1, eRF3, UPF1, UPF2, UPF3, SMG1, and EJC). SMG1 phosphorylates UPF1, leading to eRF1/eRF3 release and the formation of the decay‐inducing complex (DECID), which includes SMG5, SMG6, and SMG7 [49]. SMG7 facilitates mRNA degradation through exonucleolytic pathways, while SMG7‐mediated dephosphorylation of UPF1 enables NMD factor recycling [50] (Table 2).

**TABLE 2:** Comprehensive analyses of target, mechanism of action, toxicity, and clinical trials for each NMD inhibitor were conducted.

Molecule	Target	Mechanism of action	Toxicity	Clinical trial
Pateamine A_[16]_	UPF1‐EJC complex	Keeping eIF4AIII in a closed conformation	High toxicity	N/A
Caffeine_[63]_	SMG1	Prevention of UPF1	Low toxicity	N/A
Warfarin_[63]_				N/A
ELX‐02_[64]_		Phosphorylation	Low toxicity	N/A
NMDI‐1_[63]_	UPF1	Block of the interaction between UPF1 and SMG5	Low toxicity	N/A
NMD‐14_[63]_	SMG7		Low toxicity	N/A
5‐Azacytidine_[3]_	NMD factors	Affecting NMD factors expression by demethylation and histone acetylation	High toxicity	N/A
Curcumin_[21]_			Low toxicity	N/A

**FIGURE 3:**
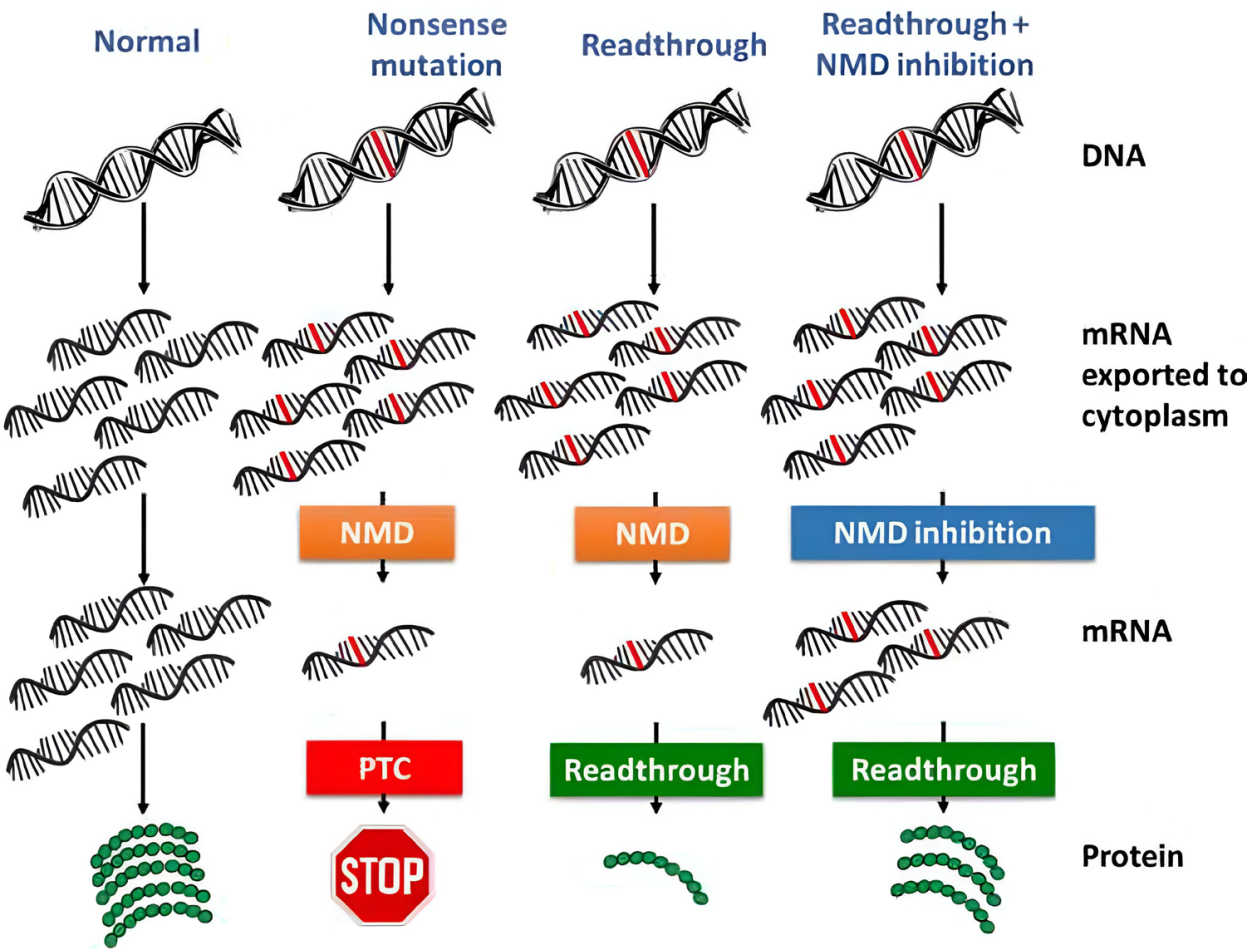
Comparison between protein syntheses from: a usually coded gene (left), a nonsense‐mutated gene through normal NMD and codon recognition (center‐left), a nonsense‐mutated gene through normal NMD and induced TR (center‐right), and a nonsense‐mutated gene with inhibited NMD and induced TR (right) [43].

Pharmacological inhibition of NMD represents a potential strategy for PTC suppression‐based therapies (Figure 3). Several compounds have been developed to target different stages of the NMD pathway. The following list resumes the most utilized molecules and mechanisms for interference with the NMD pathway:

- *Early‐stage inhibitors*: Pateamine A (PatA) prevents UPF1‐EJC binding by stabilizing eIF4AIII in a closed conformation [51].
- *General inhibitors*: Several small molecules, including caffeine, warfarin, and ELX‐02, have been reported to inhibit NMD by disrupting SMG1‐mediated phosphorylation of UPF1. Although the precise molecular mechanism of action of VG1 remains undefined, it has demonstrated robust NMD‐inhibitory activity across multiple independent studies [47, 52].
- *UPF1 stabilization*: NMDI‐1 induces the hyperphosphorylation of UPF1, leading to its localization to P‐bodies, impairs SMG5 interaction, and subsequent decay complex formation [47].
- *SMG7 inhibition*: NMD‐14, a derivative of NMDI‐1, effectively inhibits SMG7 function at low concentrations with minimal toxicity [47].
- *Indirect inhibitors*: 5‐Azacytidine and curcumin decrease NMD activity by influencing gene expression and histone acetylation [53, 54].

Despite its therapeutic potential, NMD inhibition requires careful evaluation. While beneficial in monogenic diseases and cancers with NMD‐sensitive tumor suppressor transcripts, it may also interfere with cellular surveillance mechanisms, accumulating aberrant transcripts with dominant‐negative effects. Since NMD usually silences many nonsense single‐nucleotide polymorphisms (SNPs), their unintended expression could cause adverse effects [55]. Nevertheless, targeting NMD remains a promising avenue for enhancing TR‐based therapies. A deeper understanding of NMD mechanisms and the development of novel inhibitors in combination with TRIDs could pave the way for future suppression‐based therapeutic strategies.

### 2.3 Anticodon Engineered‐tRNA (ACE‐tRNA)

**FIGURE 4:**
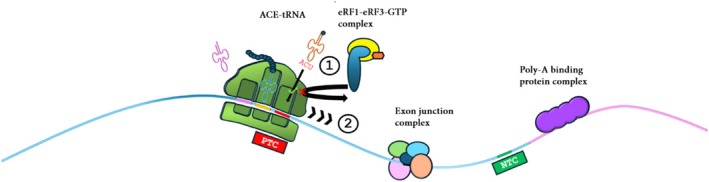
Schematic representation of ACE‐tRNA technology.

An innovative and recently developed technology promising to overcome PTCs is known as Anticodon engineered‐tRNA (ACE‐tRNA). This approach utilizes a modified aminoacyl‐tRNA with a specific anticodon to recognize a PTC. In this way, during protein translation, when a PTC occurs in the mRNA sequence, the ribosomal machinery introduces an ACE‐tRNA to continue protein synthesis, preventing the development of truncated proteins [56] (Figure 4). In the last few years, ACE‐tRNA synthesis has been significantly optimized so that it is perfectly recognized by cell ribosomal machinery for the suppression of the three PTCs [57]. Furthermore, recently, different studies have been published, proving the functionality of this approach in vitro, in CF models to restore CFTR protein [58], as well as in vivo, in mouse models with the use of adeno‐associated virus (AAVs) [59]. In particular, the Ignatova group observed the rescue of protein expression and its function with proper airway volume homeostasis [60]. Also, it was reported that ACE‐tRNAs also have an essential role in promoting the TR of PTCs in viral contexts [61].

However, discrepancies between in vitro and in vivo experiments involving ACE‐tRNAs have been observed in various studies due to differences in molecular interactions, processing pathways, and regulatory mechanisms that are present in vivo but absent or altered in vitro. This underscores the necessity of validating in vitro findings within living systems [60].

Even if the ACE‐tRNA approach yields promising results, further studies are necessary to enhance various technical features, including biosafety, bioavailability, suitable delivery systems, and, most importantly, efficacy within the natural genetic context [62].

### 2.4 Gene Editing to Rescue PTCs: Some Examples From Cystic Fibrosis

**FIGURE 5:**
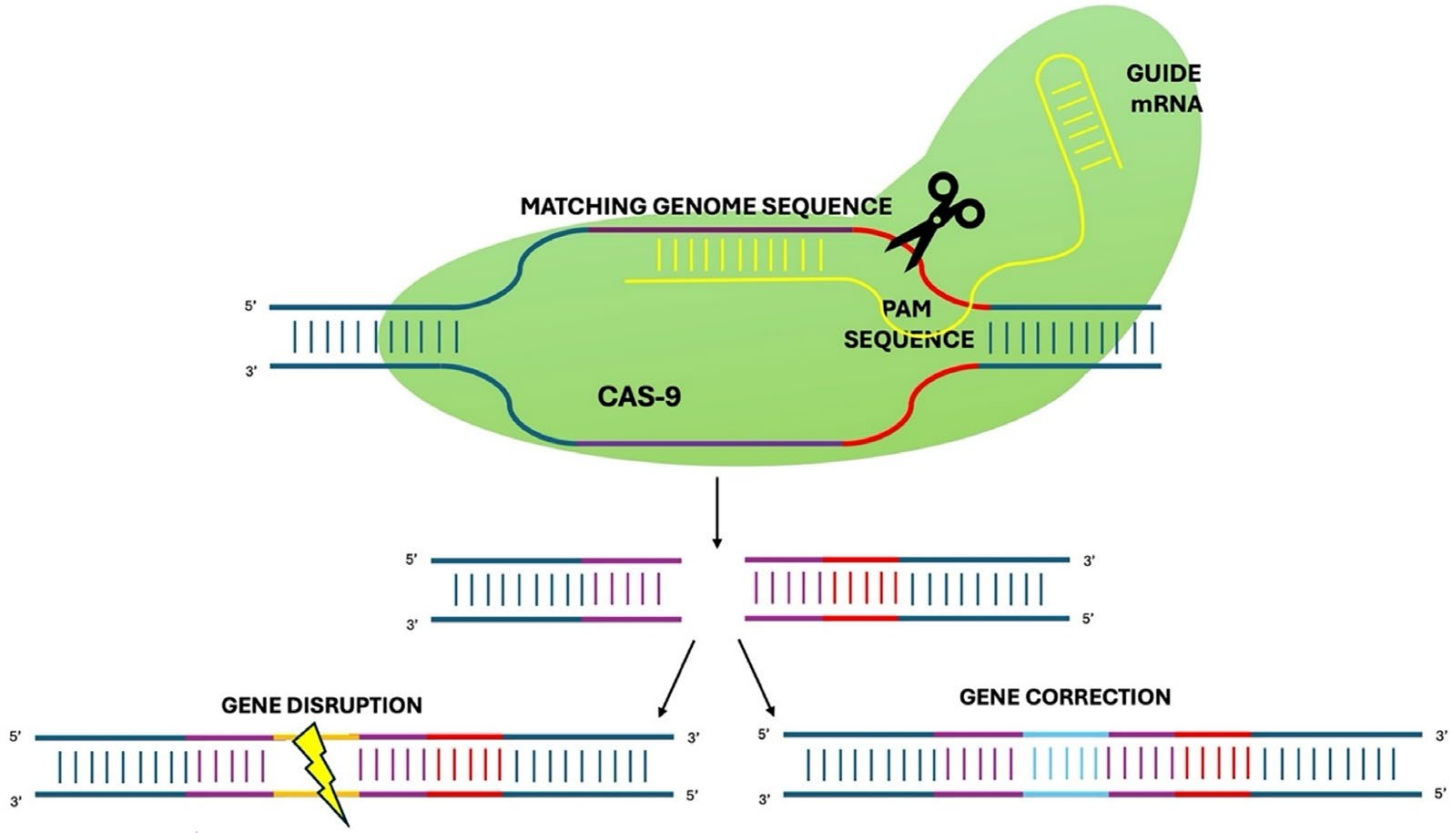
Schematic representation of gene editing based on CRISPR/Cas9 technology.

Gene editing is at the forefront of the cure of genetic diseases (Figure 5). Over the last few years, CRISPR/Cas9 technology has experienced significant growth. CRISPR/Cas9 can be used not only for nucleotide insertions or deletions (indels) but also to correct point mutations [63]. Indels can be generated by the non‐homologous end joining (NHEJ) pathway, whereas point mutation corrections can be acquired via the homology‐directed repair (HDR) pathway. Both pathways are two ways eukaryotic cells respond to DNA double‐strand breaks following the cut by the Cas9 protein [63].

Interestingly, NHEJ has successfully overcome the NMD pathway in a CF cell model. Specifically, Erwood and colleagues co‐transfected HBE cells (human bronchi), previously rendered homozygous for the W1282X stop mutation in the *CFTR* gene [64], with both *Streptococcus pyogenes* Cas9 (SpCas9) and guide RNAs (gRNAs), which targeted exons 23 and 27, respectively, downstream of the W1282X mutation and upstream of the native termination codon [65]. This gene editing generated a truncated CFTR protein, which showed significant activity after pharmacological rescue with the novel modulators VX‐661 and VX‐445 [65].

In addition to indel/point mutations, the CRISPR/Cas9 system has been recently modified to convert the A‐T base pair into the C‐G one. This modified version is known as the “adenine‐base editor” (ABE) or “base editing” and consists of a D10A Cas9 nickase fused with adenine deaminase [63]. ABE technology has been successfully applied to rescue *CFTR* nonsense mutations in a biobank of CF intestinal organoids. Indeed, Geurts and colleagues were able to correct the homozygous mutations R785X and W1282X by electroporating organoids with both a SpCas9‐ABE plasmid and a single‐guide RNA (sgRNA) plasmid, demonstrating increased forskolin‐induced swelling (FIS) [66].

Thus, ABEs can be helpful in precisely correcting a specific single‐base mutation without generating breaks in the DNA sequence. However, ABEs are only limited to base transitions. This problem has been recently overcome by creating “prime editors” (PEs) [63]. Using a Cas9 nickase fused with reverse transcriptase, PE technology can induce either transitions or transversions, indels, with no DNA double‐strand breaks [67]. PEs have been shown to rescue nonsense mutations in the *CFTR* gene. Specifically, Geurts and colleagues recently applied PEs to intestinal organoids with the *CFTR* R785X mutation, which was successfully rescued, even though ABEs displayed higher efficiency. According to the authors, PEs can potentially correct up to 419 out of 442 *CFTR* mutations in the CFTR2 database [68].

In addition, the FDA has already approved a CRISPR/Cas9 gene‐edited therapy called CTX001 (NCT05477563) to treat patients with severe sickle cell disease or beta‐thalassemia [69].

Gene therapy is a multifaceted field that requires further investigation and development. While its potential is undeniable and, for some diseases, already life‐changing, it holds the promise of being revolutionary. It represents a valuable asset in precision medicine and offers hope for treating rare mutations that cause significant genetic disorders, such as CF.

### 2.5 mRNA‐Based Therapy: Technical Aspects, New Perspectives, and Actual Ongoing Trials

The SARS‐CoV‐2 pandemic left a crucial scientific legacy: new horizons for mRNA‐based therapy applications. This approach had not been extensively explored before the COVID‐19 outbreak. Still, it has spread over the last few years, thanks also to the development of vaccines and approaches to prevent and treat some types of cancer using mRNA‐based vaccines against certain tumors [70, 71]. Despite the immediate and straightforward idea of using mRNA as a therapeutic approach, several challenges have been identified by researchers. Specifically, various aspects must be considered, including mRNA production and its associated engineering issues, its capacity to remain stable and avoid degradation, the ability to target specific tissues, potential off‐target effects, and the delivery systems [72]. Considering its chemical and structural properties, a correct mRNA design is based on critical structural features, including the 5′‐cap, the poly(A) tail, the 5′‐and 3′‐untranslated regions (UTRs), and the ORF. These are the main features that have been improved over the last few years, enabling the creation of powerful and promising mRNA‐based therapies and their optimization, which has made them suitable for entering clinical trials [72, 73].

Contemporary design enhancement and the choice of the delivery system represent a challenge. Xiao and collaborators have described all the advances for the best strategy and biomedical application of mRNA‐based therapy. Precisely, they have thoroughly elucidated non‐viral delivery systems, which include naked mRNA, protein‐mRNA complexes, lipid‐based carriers, polymer‐based carriers, or hybrid carriers, and have also explained their applications, such as functional protein expression for protein replacement therapy [74]. Achieving the correct target organ is a crucial final goal in mRNA‐based therapy approaches. To overcome this issue, a new method, named Selective Organ Targeting (SORT) nanoparticles, has been recently developed by Cheng et al. for the administration of both mRNA and CRISPR/Cas9 technology [75]. However, other issues have arisen regarding the target organ. For instance, the thick mucus layer in airway epithelia is a critical concern in CF. As regards this aspect, in 2020, Sanchez et al. and Sahu et al. briefly explained the problems to overcome in this specific case, which is not only related to the presence of a viscous mucus but also to the clearance activity of phagocytes, with the ability to avoid any intracellular control system that can recognize the mRNA as exogenous and eliminate it and the event of the proper translation into the functional protein [76, 77].

However, some drugs have undergone thorough preclinical studies, including in vitro and in vivo models, and have progressed to clinical trials in recent years. This is true for CF, Fabry disease, Duchenne muscular dystrophy, choroideremia, cancer, and other pathological conditions. For example, in collaboration with Moderna, three companies have developed new approaches to treat severe pathologies, including NRDs, particularly Arcturus Therapeutics, ReCode Therapeutics, and Vertex Pharmaceuticals. Arcturus Therapeutics has patented a delivery system named LUNAR and two drugs, ARCT‐032 and ACTR‐810 (NCT05712538), which have achieved Phase I clinical trials with promising results for safety, tolerability, and kinetics [78] (https://arcturusrx.com/rna‐mrna‐proprietary‐technologies/). ReCode Therapeutics has developed SORT lipid nanoparticles (LNPs) with promising in vitro results for CF [79]. In collaboration with Moderna, Vertex Pharmaceuticals has developed VX‐552, which is currently in Phase I clinical trials for safety evaluation (NCT05668741).

## 3 Conclusion

Nonsense mutations pose a significant challenge in the treatment of NRDs, often resulting in severe phenotypes due to the loss of functional proteins. However, recent advances in therapeutic strategies such as TRIDs, NMD inhibition, ACE‐tRNAs, gene editing, and mRNA‐based therapies offer promising avenues for restoring protein expression and function, with new approaches continuing to emerge [80]. Despite encouraging preclinical and clinical results, further research is necessary to gain a deeper understanding of nonsense suppression biology. Several challenges remain, including optimizing drug efficacy, minimizing off‐target effects, and enhancing targeted delivery systems. Future research should focus on refining these approaches and exploring integrated and personalized treatments to improve therapeutic outcomes and ensure continuous improvement in the quality of care.

## Conflicts of Interest

The authors declare no conflicts of interest.
